# Altered biodistribution of [^68^Ga]Ga-DOTA-TOC during somatostatin analogue treatment

**DOI:** 10.1007/s00259-024-06659-0

**Published:** 2024-02-26

**Authors:** T. van de Weijer, F. Bemer, J. de Vos-Geelen, B. Hermans, C. Mitea, J. A. J. van der Pol, T. Lodewick, J. E. Wildberger, F. M. Mottaghy

**Affiliations:** 1 Department of Radiology and Nuclear Medicine, ENETS Center of Excellence, Maastricht University Medical Center (MUMC+), P. Debeylaan 25, P.O. Box 5800, 6202, 6229 HX AZ Maastricht, The Netherlands; 2 School of Nutrition and Translational Research in Metabolism (NUTRIM), University of Maastricht (UM), P. Debeylaan 25, P.O. Box 5800, 6202, 6229 HX AZ Maastricht, The Netherlands; 3 Department of Medical Oncology, ENETS Center of Excellence, MUMC+, P. Debeylaan 25, P.O. Box 5800, 6202, 6229 HX AZ Maastricht, The Netherlands; 4 School for Oncology and Reproduction (GROW), UM, P. Debeylaan 25, P.O. Box 5800, 6202, 6229 HX AZ Maastricht, The Netherlands; 5 School for Cardiovascular Diseases (CARIM), UM, P. Debeylaan 25, P.O. Box 5800, 6202, 6229 HX AZ Maastricht, The Netherlands; 6 Department of Nuclear Medicine, University Hospital RWTH Aachen University, Pauwelsstr. 30, 52074 Aachen, Germany

**Keywords:** ^68^Ga-DOTATOC, NENs, Biodistribution, Somatostatin receptor analogue therapy

## Abstract

**Purpose:**

The need for an interval between the administration of long-acting Somatostatin Receptor Analogues (SSA) and the [^68^Ga]Ga-DOTA-TATE PET has been questioned based on recent literature in the new EANM guidelines. Here an earlier studies showed that SSA injection immediately before SSTR PET had minimal effect on normal organ and tumor uptake (1). However, data are scarce and there are (small) differences between [^68^Ga]Ga-DOTA-TATE and [^68^Ga]Ga-DOTA-TOC binding affinity, and it remains unknown whether these findings can be directly translated to scans with [^68^Ga]Ga-DOTA-TOC as well. The purpose of this study was to assess the effect of SSA use on the biodistribution in a subsequent [^68^Ga]Ga-DOTA-TOC PET/CT and compare this intra-individually across several cycles of SSA treatments.

**Methods:**

Retrospectively, 35 patients with NENs were included. [^68^Ga]Ga-DOTA-TOC PET at staging and after the 1st and 2nd cycle of SSA were included. SUVmean and SUVmax of blood, visceral organs, primary tumor and two metastases were determined. Also, the interval between SSA therapy and the PET scan was registered.

**Results:**

Treatment with SSA resulted in a significantly higher bloodpool activity and lower visceral tracer uptake. This effect was maintained after a 2nd cycle of SSA therapy. Furthermore, there was an inverse relationship between bloodpool tracer availability and visceral tracer binding and a positive correlation between bloodpool tracer availability and primary tumor tracer uptake. With an interval of up to 5 days, there was a significantly higher bloodpool activity than at longer intervals.

**Conclusion:**

Absolute comparison of the SUV on [^68^Ga]Ga-DOTA-TOC PET should be done with caution as the altered biodistribution of the tracer after SSA treatment should be taken into account. We recommend not to perform a scan within the first 5 days after the injection of lanreotide.

**Supplementary Information:**

The online version contains supplementary material available at 10.1007/s00259-024-06659-0.

## Introduction

Neuroendocrine neoplasms (NEN) are a heterogeneous group of malignancies with a neuroendocrine phenotype, i.e., expressing markers of neuroendocrine differentiation [1]. Although the incidence of these types of neoplasms is low, NENs represent 2% of all malignancies and the incidence is rising [2]. A better follow-up and treatment of these patients becomes more important.

The majority of NENs are characterised by an expression of the somatostatin receptor (SSR). The imaging and follow-up of low-grade NENs is preferably done implementing SSR imaging using PET [3, 4]. Of the available tracers, the ^68^Ga-labeled somatostatin analogues, [^68^Ga]Ga-DOTA-TATE and [^68^Ga]Ga-DOTA-TOC are the most widely used [5–7]. SSR-targeted somatostatine ligand analogues used for (cold) treatment (non-radio-active ligand treatment with somatostatin analogues (SSA)) or Peptide Receptor Radionuclide Therapy (PRRT) bind at the same Somatostatin receptor site (SSR) as [^68^Ga]Ga-DOTA-TATE and [^68^Ga]Ga-DOTA-TOC, rendering this theranostic approach well established [8–16]. However, the influence of especially the cold compound treatment on imaging quality remains under discussion. Recently, the EANM and SNMMI guidelines for SSR imaging have been revised [17]. Here, it was suggested that an interval of 3–4 weeks after administration of long-acting SSA to avoid potential SSR blockade might be unnecessary. This is based on the study of Aalbersberg et al. [18], who showed that lanreotide (somatuline) treatment immediately before SSTR PET had minimal effect on normal organ and tumor uptake. However, data on this subject are scarce and these studies have been mainly performed with ^68^Ga-[^68^ Ga]Ga-DOTA-TATE.

There are five subtypes of SSR of which the SSR_2_ and SSR_5_ are most often expressed in NENs. These receptors are utilised for both imaging as well as treatment of NENs [19–21]. The pharmacokinetics of DOTATOC and [^68^Ga]Ga-DOTA-TATE were found to be comparable [22]. However, there are some small differences. For one, [^68^Ga]Ga-DOTA-TATE has a binding affinity more than nine fold higher for SSR_2_ in comparison with [^68^Ga]Ga-DOTA-TOC [23]. A difference in uptake was found for these tracers with a maximal uptake of [^68^Ga]Ga-DOTA-TATE in Gastro-Entero-Pancreatic NENs (GEP-NENs) with higher binding affinity of [^68^Ga]Ga-DOTA-TATE than for [^68^Ga]Ga-DOTA-TOC imaging [5]. Although the diagnostic value of PET/CT with [^68^Ga]Ga-DOTA-TATE and [^68^Ga]Ga-DOTA-TOC in the same patients with GEP-NENs is comparable, the differences between the tracer based on the effects of SSA on the biodistribution of these tracers remains unknown.

All previous studies [24–28] have shown that octreotide treatment alters the degree of SSR expression in NETs. For [^68^Ga]Ga-DOTA-TATE, some studies have shown that SSR-targeted treatment does alter biodistribution by lowering tracer uptake in the visceral organs, though it does not seem to affect tracer uptake in the primary tumor or its metastases [18, 29, 30]. The effect does depend on the analogue used (short vs. long-acting SSA). Furthermore, acute administration within 24 h before the scan will result in dramatically lower [^68^Ga]Ga-DOTA-TATE binding affinity in the visceral organs and slightly increased tracer binding in neoplasms [18]. The current procedure standard/practice guideline is still not explicit on a recommendation of withdrawal since prospective data are only available for lanreotide and not yet octreotide acetate LAR [17]. Limited data are available regarding the long-term effects of treatment on tracer affinity [31, 32]. Furthermore, the current studies are all based on findings gained using [^68^Ga]Ga-DOTA-TATE as a ligand. As there are (small) differences between [^68^Ga]Ga-DOTA-TATE and [^68^Ga]Ga-DOTA-TOC binding affinity, it remains unknown whether the conclusions based on these findings can be directly translated to scans with [^68^Ga]Ga-DOTA-TOC as well. Also, it remains unknown whether the internalisation of the SRR after treatment with a SSA is maintained after a second or third cycle of treatment. This makes the comparison of follow-up scans with variable SSR expression in the tumours and organs difficult to interpret. Therefore, the aim of this study was to assess the effect of clinical long-acting somatostatin analogue use on the uptake of [^68^Ga]Ga-DOTA-TOC intra-individually. Additionally, we assessed whether the interval between SSA application and PET acquisition did affect the SSR expression as measured by means of SUV.

## Methods

### Patients

The study was set-up as a retrospective cohort study. All patients diagnosed with a locally advanced or metastatic GEP-NEN at the Maastricht University Medical Center (MUMC +) that underwent a [^68^Ga]Ga-DOTA-TOC PET scan at either the Uniklinik Aachen or at the Maastricht University Medical Center (MUMC +) between 2019 and 2021 were included. Only patients treated with lanreotide were included. The scan after therapy had to be performed within 2 months after the last lanreotide injection. Furthermore, patients needed to have a baseline [^68^Ga]Ga-DOTA-TOC scan prior to initiation of SSA therapy as well as for follow-up. All following tumour evaluations were included if available. The study was approved by the Medical Ethical Committee according to the declaration of Helsinki and registered under METC-number 2021–2992.

Patient characteristics were gathered from the digital patient files including primary tumour type and site, age, length and weight at the moment of the scan. Furthermore, the interval between lanreotide injection and the scan was registered.

### Study protocol

At Maastricht UMC + site patients were scanned on a 5 ring PET system (GE, Discovery MI, Milwaukee, Michigan, United States). At the Uniklinik Aachen, patients were scanned on a 16 PET/CT system (Gemini TF, Philips Medical Systems, Best, the Netherlands). All scans were performed with a standardized protocol, administrating 1.5 MBq/kg of [^68^Ga]Ga-DOTA-TOC 40 min prior to the scan (range 30–45 min), with 2–2.5 min per bed position. All data was reconstructed according to standardized protocol using iterative reconstruction.

A low-dose CT scan was acquired for attenuation correction and anatomical correlation. Subsequent to the PET acquisition, diagnostic CT images were acquired with a chest CT and the upper abdomen in the arterial phase and a full abdominal CT in the portal venous phase after intravenous administration of iopromide 300 (Ultravist, Bayer Healthcare, Berlin, Germany).

Visual reading was performed by a senior nuclear medicine physician (10 years of experience with [^68^Ga]Ga-DOTA-TOC PET/CT) and a research assistant trained for the project. Maximum and mean standardized uptake values (SUVmax and SUVmean) were quantified in a spherical volume of interest (VOI) of 2 ml at the primary tumor (if applicable), 2 metastases, bloodpool at the height of the aortic arch, spleen, liver and the uncinate process of the pancreatic tail. In the follow-up scans, the VOI was placed in the same region as in the initial scan.

### Statistical analysis

Normal distribution of the data was evaluated with the Shapiro–Wilk test. For image quantification, paired *t*-tests were performed to identify significant differences in uptake of [^68^Ga]Ga-DOTA-TOC between the scans. Also, linear regression analysis was performed using *R*^2^ and Pearson correlation coefficients. Data are expressed as mean ± standard deviation (SD), with a *p*-value of < 0.05 considered significant. Analyses were performed in SPSS (version 28, IBM, USA).

## Results

### Patient population

In total, 35 patients were included, of which 54% were male (*n* = 19). Mean age at primary staging was 62.6 ± 9.6 years (Table 1). There were 5 patients with a primary NEN of the pancreas, 23 with a NEN of the gastro-intestinal tract and 7 patients had a gastro-intestinal type NEN of unknown origin.

**Table 1:** Patient characteristics

Number of patients (male/female)	35 (19/16)
Age (years)	62.6 ± 9.6
Primary tumor	
- Pancreas	*N* = 5
- Gastro-intestinal	*N* = 23
- Unknown origin	*N* = 7
Mean follow-up interval (months)	
- Between baseline and FUP1	15.9 ± 18.4 (*n* = 35)
- Between FUP1 and FUP2	10.4 ± 5.5 (*n* = 19)

Mean follow-up interval between baseline and FUP1 was 15.9 ± 18.4 months and between FUP1 and FUP2 was 10.4 ± 5.5 months. In 19 patients, a second scan was performed after prolonged lanreotide treatment (FUP2; *n* = 19). In some of these patients, also a 3rd and 4th follow-up scan was performed after therapy. However, the number of these patients was not sufficient to perform meaningful statistical analyses regarding the additional scans.

### Biodistribution of [^68^Ga]Ga-DOTA-TOC before and after lanreotide treatment

**Fig. 1:**
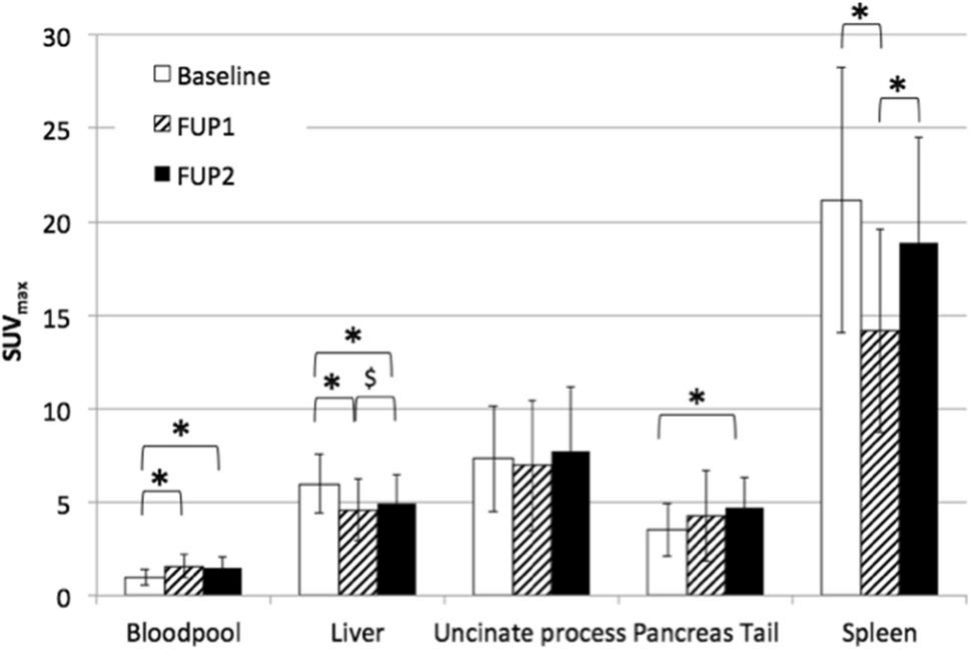
SUVmax depicted for the bloodpool and visceral organs, *p < 0.05 and $*p* < 0.10

Upon lanreotide treatment, the tracer availability in the bloodpool increased significantly (SUVmax baseline 0.99 ± 0.44, FUP1 1.57 ± 0.62, FUP2 1.51 ± 0.56, *p* < 0.05, see Table 2). This was accompanied by a decreased tracer accumulation in the liver (SUVmax baseline 5.97 ± 1.59, FUP1 4.58 ± 1.66, FUP2 4.95 ± 1.55, *p* < 0.05) and spleen (SUVmax baseline 21.17 ± 7.11, FUP1 14.18 ± 5.46, FUP2 18.85 ± 5.68, *p* < 0.05, see Table 2). There was a tendency towards an incline in tracer accumulation in spleen and liver between FUP1 and FUP2; however, this characteristic was only significant in the spleen. The accumulation in the uncinate process was variable, and not significantly different between baseline and follow-up scans. Interestingly, the pancreas tail showed an increase in tracer accumulation during treatment, an effect that was maintained in the second follow-up period (baseline 3.50 ± 1.40, FUP1 4.38 ± 2.48 and FUP2 4.70 ± 1.65, *p* < 0.05, Fig. 1). The results of the statistical analysis of SUVmean appeared similar to SUVmax, and in particular significant differences in biodistribution remained unchanged (see supplemental information).

**Table 2:** SUVmax (mean ± SD) for the bloodpool and visceral organs at baseline and after treatment at FUP1 and FUP2

	Baseline (*n* = 35)	FUP1 (*n* = 35)	FUP2 (*n* = 19)
Bloodpool	0.99 ± 0.44	1.57 ± 0.62*	1.51 ± 0.56*
Liver	5.97 ± 1.59	4.58 ± 1.66*	4.95 ± 1.55*^$^
Uncinate process	7.32 ± 2.84	6.95 ± 3.50	7.75 ± 3.39
Pancreatic tail	3.50 ± 1.40	4.38 ± 2.48	4.70 ± 1.65*
Spleen	21.17 ± 7.11	14.18 ± 5.46*	18.85 ± 5.68*

*p < 0.05 and $*p* < 0.10

**Fig. 2:**
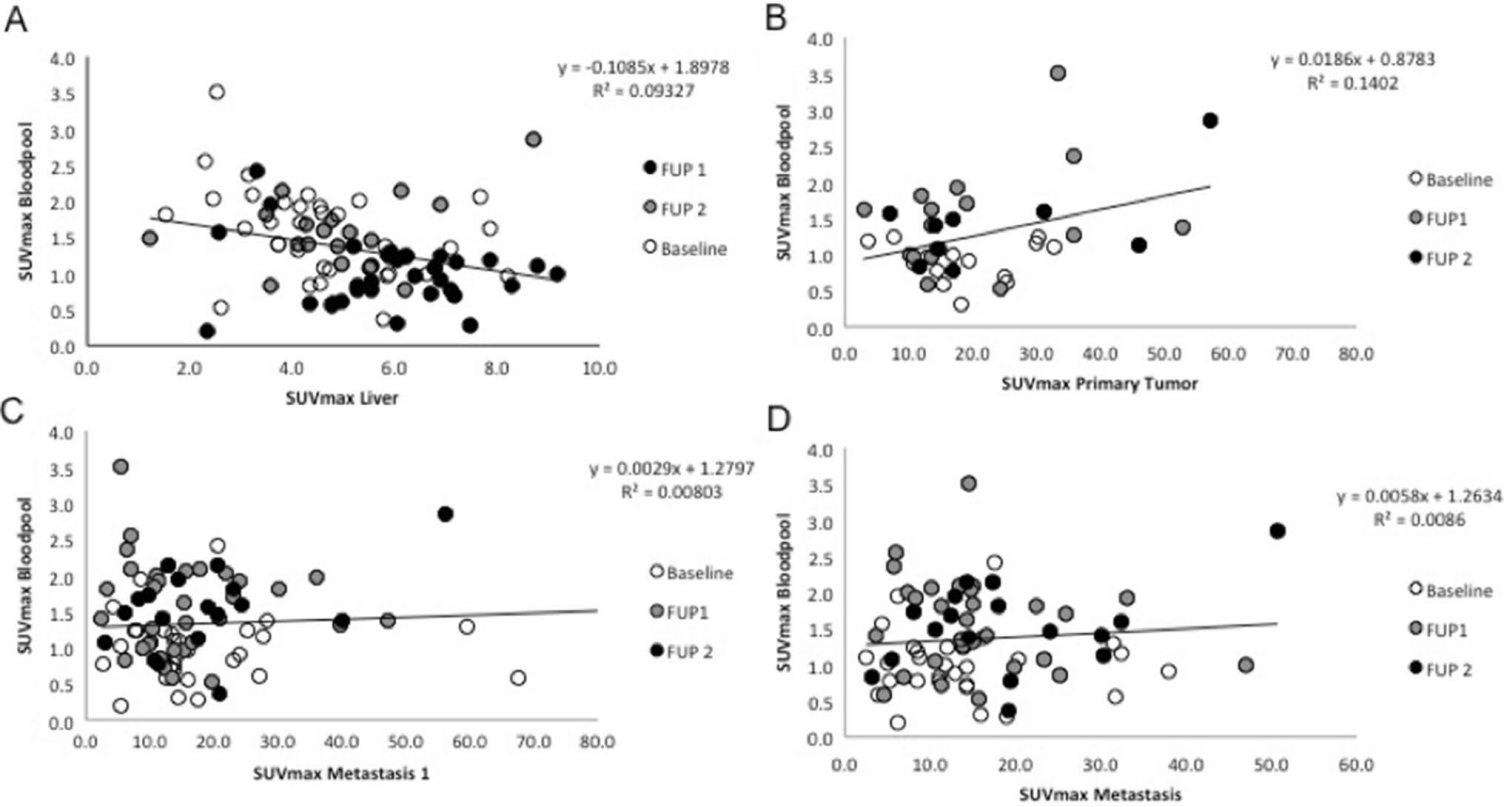
Scatter plot of the correlation of the bloodpool with the organs and tumor locations. In **A**, the bloodpool and Liver SUVmax show an inverse correlation (*p* = 0.012, *n* = 35). In panel **B**, the bloodpool and primary tumor SUVmax show a positive correlation (*p* = 0.012, *n* = 13). The metastasis (**C** and **D**) shows a similar trend towards a positive correlation with bloodpool activity as the primary tumor, however this was not a significant finding (*p* > 0.05, *n* = 35)

A relationship between plasma tracer availability in the bloodpool and liver tracer binding was found using linear regression analysis. Here the correlation coefficient was − 0.1085, a *R*^2^ of 0.0937 indicating a small though significant (*p* = 0.012) effect. This is also depicted in Fig. 2. The plasma tracer availability showed no significant correlation for the other visceral organs.

### [^68^Ga]Ga-DOTA-TOC binding in primary tumor and metastases

In only 13 patients, the primary tumor was still in situ. In all patients, at least 2 metastases could be measured (*n* = 35). The tumor lesions did not show significant difference in tracer uptake, when compared to baseline. Also, there was no significant difference between FUP1 and FUP2 (see Table 3). There was a significant correlation between bloodpool tracer availability and tracer uptake in the primary tumor in this cohort with a correlation coefficient of 0.7543, a *R*^2^ of 0.1402 and *p* = 0.008 (*n* = 13, see Fig. 2B). The metastases did show a similar tendency; however, this was not significant (*n* = 35, Fig. 2C and D).

**Table 3:** SUVmax (mean ± SD) for the primary tumor and 2 metastasis treatment at FUP1 and FUP2. There are no significant differences in SUVmax during follow-up, when compared to the baseline

	Baseline (*n* = 35)	FUP1 (*n* = 35)	FUP2 (*n* = 19)
Primary tumor	18.42 ± 8.57	20.48 ± 13.36	23.90 ± 17.22
Metastasis 1	17.12 ± 13.63	19.99 ± 25.85	18.04 ± 12.41
Metastasis 2	13.97 ± 9.19	14.96 ± 9.15	18.99 ± 11.75

### Interval of lanreotide administration and the [^68^Ga]Ga-DOTA-TOC PET scan

**Fig. 3:**
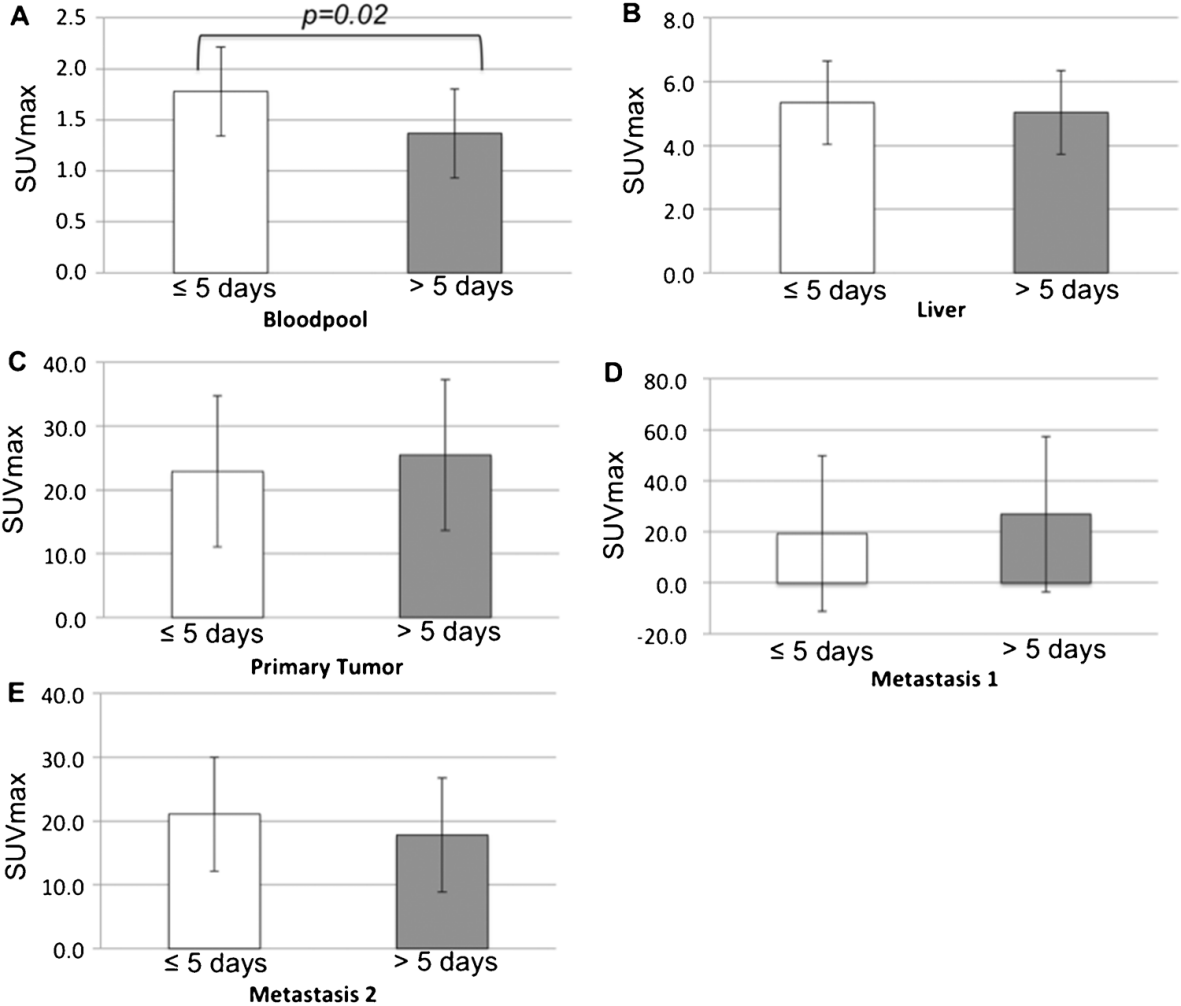
SUVmax of the bloodpool (**A**), Liver (**B**), Primary tumor (**C**), Metastasis 1 (**D**) and Metastasis 2 (**E**) of patients were the scan was performed either within 5 days (white bar) or after 5 days (gray bar) of lanreotide injection. These data show a significant higher bloodpool activity in patients that had a scan within 5 days after injection when compared to injection more than 5 days before the scan, *p* = 0.02

**Fig. 4:**
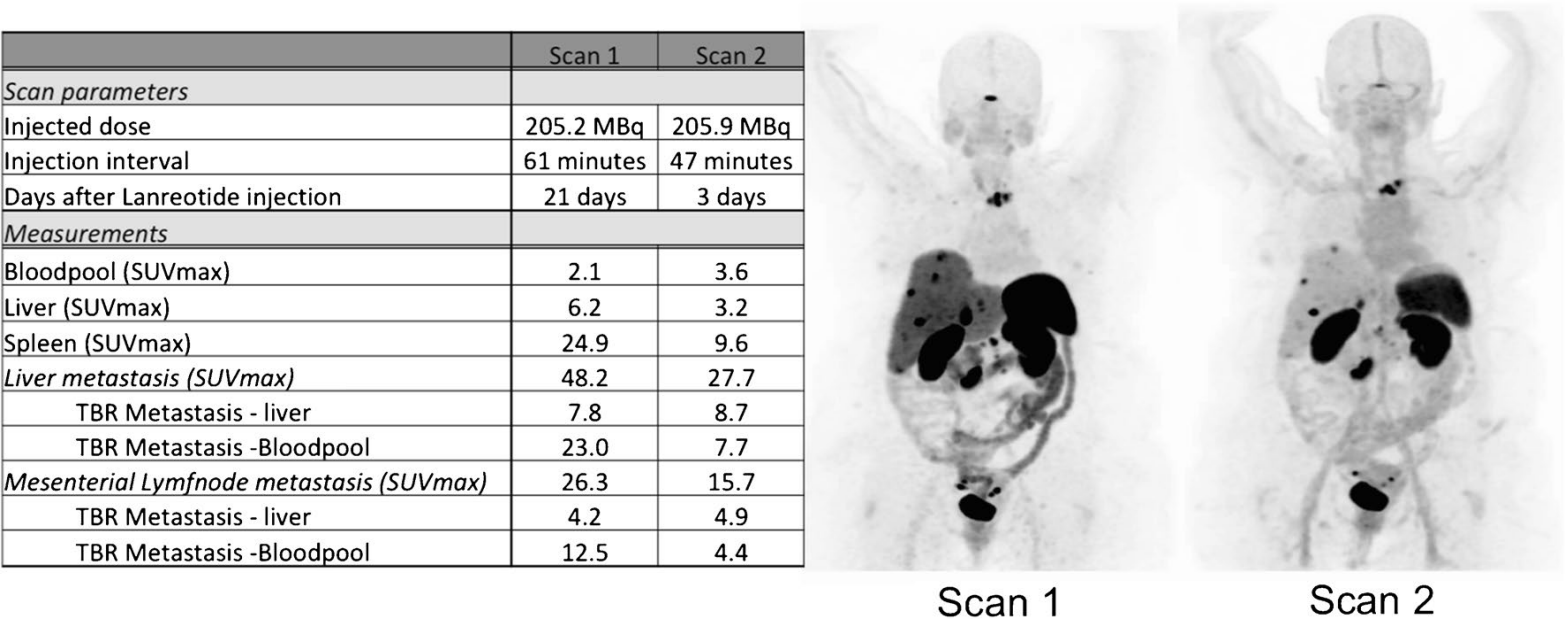
Example of a patient scanned twice after lanreotide treatment with first follow-up scan 21 days after lanreotide injection and the second scan after 3 days of lanreotide injection (interval between the scans is approximately 6 months). A visual and quantifiable difference between scans can be found in visceral and bloodpool availability of the tracer, in this case resulting in a difference in tumor uptake of the tracer

The interval between administration of lanreotide and the PET scan was registered for all 35 scans. Here the average time of lanreotide injection before the scan was 14.01 ± 9.0 days. To investigate the effects of the time-interval on the biodistribution, two groups of patients were considered depending on the time of lanreotide injection (within 5 days or more than 5 days prior to the scan). There was a significantly higher tracer availability within the blood when the tracer was injected within 5 days prior to the scan (SUVmax bloodpool < 5 days 1.78 ± 0.48 vs > 5 days 1.37 ± 0.44, *p* = 0.02, see Fig. 3). However, this effect was not significant for the visceral organs or the tumours (Fig. 4). Here, a patient was scanned twice after treatment with lanreotide after 21 days of injection (follow-up 1) and a half year later after 3 days of lanreotide injection (follow-up 2). In the same subject, the intensity of the tracer accumulation in the background was lower at 3 days after injection (also quantified in Fig. 4), when compared to 21 days. Liver somatostatin receptor binding intensity was half the intensity after 3 days, when compared to 21 days after injection, also resulting in higher tracer availability in the blood. Tumor intensity on the scan was slightly higher, but normalized after correction of liver accumulation. Other parameters were similar.

## Discussion

This study demonstrates that the visceral binding of [^68^Ga]Ga-DOTA-TOC is significantly reduced after lanreotide treatment. This is in line with previous studies focusing on [^68^Ga]Ga-DOTA-TATE. Despite small differences in binding affinity between these two tracers [23], the effect of somatostatin receptor analogue treatment on the biodistribution of the tracers seems comparable. Both in our study with [^68^Ga]Ga-DOTA-TOC as well as in the study of Ayati et al. [29] with [^68^Ga]Ga-DOTA-TATE, it was shown that that long-acting somatostatin analogue treatment diminishes radio-active ligand uptake in the liver, spleen, and thyroid but did not compromise tracer uptake in residual primary tumor and metastatic lesions. Also, the study of Cherk et al. [33] showed that after SSA therapy spleen and liver SUVmax on [^68^Ga]Ga-DOTA-TATE imaging decreased significantly. Hence, the effects for both ligands are comparable.

Interestingly, the effect of this alteration in biodistribution is maintained after a second follow-up scan during somatostatin analogue therapy. We did not find a rebound effect with upregulation of somatostatin receptors or a desensibilisation of the somatostatin analogue therapy during treatment, resulting in a similar tracer distribution with lowered visceral binding of the [^68^Ga]Ga-DOTA-TOC ligand. However, the binding affinity in the liver and spleen seems to slightly increase during the second follow-up period, although it is still lower than pre-treatment. Nonetheless, there may be a habituation effect after longer treatment periods. This has not been reported before and is relevant as it remains unclear to what extent the effect of therapy may remain stable and whether differences in uptake during follow-up are caused by disease response rather than being linked to duration of treatment.

Tumor uptake did correlate with bloodpool availability of the tracer; however, we did not observe changes in absolute uptake in the organs or tumor lesions. More importantly, there was a significant correlation between tracer availability in the bloodpool and visceral and tumor uptake. Possibly our study population was too small to pick up significant differences in tissue and tumor uptake. Also, tumor uptake may be variable as therapy response may vary amongst patients, obscuring the therapy effects. Therefore, we do feel there is an urge for standardization of administration protocols of long-acting Somatostatin receptor analogues before [^68^Ga]Ga-DOTA-TOC scans. Current EANM guidelines recommend withdrawal of somatostatin analogues for 3–4 weeks prior to PET imaging [31, 32, 34]. However, this may be on the safe side, as in the current study the effects of alterations in tracer distribution seem to diminish after 5 days of treatment. Ayati et al. [29] reported that long-acting octreotide treatment diminished [^68^Ga]Ga-DOTA-TATE uptake in the liver, spleen, and thyroid but did not compromise tracer uptake in residual primary tumor and metastatic lesions. This is in line with the findings of this study. Here, the average interval was 25.1 ± 14.8 days, indicating that the pool of patients with scans within 5 days of octreotide administration in this study was low and the effect of administration within 1 week prior to the scan was not separately assessed in this study. The study of Aalbersberg et al. [18] does show that these acute effects may be anticipated for [^68^Ga]Ga-DOTA-TATE. The results of our study suggest that this effect is similar for [^68^Ga]Ga-DOTA-TOC PET. Hence, for [^68^Ga]Ga-DOTA-TOC PET scans, a recommendation of an interval application of long-working somatostatin analogues of at least 5 days should be sufficient. Nonetheless, small differences in biodistribution may be anticipated even after 5 days of administration of the SSA. Interpreting absolute values in the analysis of [^68^Ga]Ga-DOTA-TOC PET should be done with caution. The study of Cherk et al. [33] that showed that long-acting SSA therapy decreases [^68^Ga]Ga-DOTA-TATE uptake in the thyroid, spleen and liver and in most cases increases intensity of uptake within metastases. They pointed out that this indeed has significant implications for the interpretation of the PET/CT following the commencement of therapy as increased intensity alone may not represent true progression. Therefore, the measurement of tumor-to-liver ratios may be considered when performing quantitative analysis.

Standardization of the imaging protocol after injection of the SSA would be preferable; however, in clinical practice may be complicated. The interval between the scans is variable for the different tumor types, size and grade of the tumour. According to the European Neuroendocrine Tumor Society (ENETS) for instance, for Neuroendocrine colorectal cancers, R0 resection of a rectal NET grade 1 with local lymph node metastasis or grade 2/3 smaller than 10 mm, a [^68^Ga]-somatostatin receptor PET/CT is recommended initially and after 12 months [20]. For non-resected metastatic neuroendocrine neoplasmata of the pancreas, imaging should be performed at 3 months and then every 3–6 months for 2 years [35]. This is also variable for different NENs and highly depending on clinical parameters and plasma CgA levels. It is important to note that the optimal timing for post-therapy imaging may be influenced by the specific treatment plan prescribed by the healthcare provider. Therefore, the decision on when to perform the follow-up scan should be made in consultation with the treating physician, the dosimetry expert radiation physicist, taking into account the individual patient’s clinical condition and response to therapy.

A limitation of the study was the small sample size. Only 35 patients could be included and in only 19 patients a second follow-up was performed. Also, the study was set-up as a retrospective cohort study. For further confirmation of the current study findings, more research in greater populations and prospective studies are needed.

In conclusion, absolute comparison of the SUV in lesions on [^68^Ga]Ga-DOTA-TOC PET should be done with caution as the altered biodistribution of the tracer after somatostatin analogue treatment should be taken into account. We recommend not to perform a scan within the first 5 days after injection of lanreotide. Furthermore, tumor-to-liver ratios may be considered when performing quantitative analysis in clinical practice.

### Supplementary Information

Below is the link to the electronic supplementary material.

Supplementary file1 (PNG 50 KB)

## Data Availability

The datasets generated during and/or analysed during the current study are available from the corresponding author on reasonable request.
