# Supplementary figures and images for: Altered biodistribution of [68Ga]Ga-DOTA-TOC during somatostatin analogue treatment
Source: Eur J Nucl Med Mol Imaging. 2024 Feb 26;51(8):2420–7. doi: 10.1007/s00259-024-06659-0 (PMC11178651; doi:10.1007/s00259-024-06659-0)

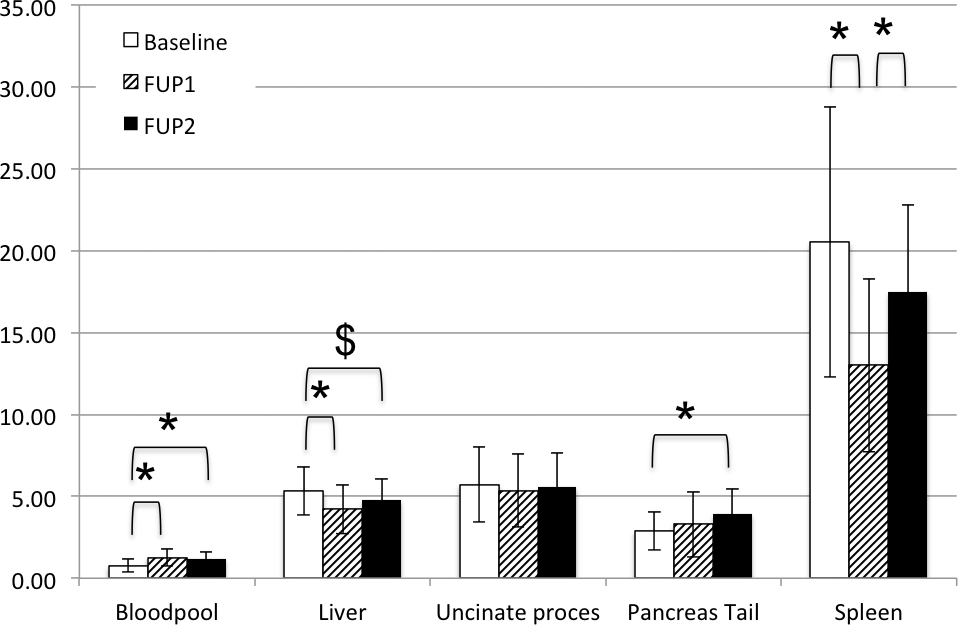

Supplement: Supplementary file 1 — Supplementary file1 (PNG 50 KB) [file 259_2024_6659_MOESM1_ESM.png]
